# Role of protein arginine methyltransferase 5 over-expression in HTLV-1-driven cellular transformation and leukemia

**DOI:** 10.1186/1742-4690-11-S1-O65

**Published:** 2014-01-07

**Authors:** Amanda R Panfil, Jacob J Al-Saleem, Robert A Baiocchi, Patrick L Green

**Affiliations:** 1 Center for Retrovirus Research, Department of Veterinary Biosciences, Comprehensive Cancer Center and Solove Research Institute, The Ohio State University, Columbus, OH, USA; 2 Division of Hematology-Oncology, Department of Internal Medicine, College of Medicine, The Ohio State University, Columbus, OH, USA

## 

Human T-cell leukemia virus -1 (HTLV-1) is a delta retrovirus that infects an estimated 15-25 million people worldwide. HTLV-1 is the causative infectious agent of adult T-cell leukemia/lymphoma (ATL) and a neurodegenerative disease (HAM/TSP). While the probability of presenting any symptoms related to HTLV-1 infection is relatively low (roughly 5-10% for the lifetime of an infected individual), the disease progression and prognosis of those infected individuals who develop ATL is fatal, with a median survival range of 8-10 months. Unfortunately, ATL is highly chemotherapy resistant and while many current therapies improve ATL patient survival, the patients consistently relapse. Therefore, a need exists to develop treatments that improve ATL outcome. We have recently identified PRMT5 (protein arginine methyltransferase 5) as a potential target to modulate HTLV-1 gene expression. We find that PRMT5 protein levels are elevated in T-cell leukemia/lymphoma cell lines compared to freshly isolated naïve T-cells. PRMT5 RNA levels do not correlate to PRMT5 protein levels, suggesting a possible post-transcriptional method of regulation. Furthermore, we also show that PRMT5 protein expression is slightly elevated during short-term immortalization, but gains highest expression after transformation and IL-2 independence. Utilizing shRNA vectors, we demonstrate that knockdown of endogenous PRMT5 results in a decrease in viral p19 production in a HTLV-1-transformed cell line. Finally, we observe a decrease in cell proliferation and in viral gene expression when HTLV-1-infected/-transformed cells are treated with a novel small molecule inhibitor of PRMT5. In conclusion, we find PRMT5 to be a positive regulator of HTLV-1 gene expression.

